# Extracellular vesicles-associated tRNA-derived fragments (tRFs): biogenesis, biological functions, and their role as potential biomarkers in human diseases

**DOI:** 10.1007/s00109-022-02189-0

**Published:** 2022-03-24

**Authors:** Qiuyan Weng, Yao Wang, Yaoyao Xie, Xiuchong Yu, Shuangshuang Zhang, Jiaxin Ge, Zhe Li, Guoliang Ye, Junming Guo

**Affiliations:** 1 Department of Gastroenterology, The Affiliated Hospital of Medical School, Ningbo University, Ningbo, 315020 China; 2 Department of Biochemistry and Molecular Biology and Zhejiang Key Laboratory of Pathophysiology, Ningbo University School of Medicine, Ningbo, 315211 China; 3 Institute of Digestive Diseases of Ningbo University, Ningbo, 315020 China

**Keywords:** tRNA-derived fragments (tRFs), Biological functions, Extracellular vesicles (EVs), Biomarker, Cancer

## Abstract

Traditionally, transfer RNAs (tRNAs) specifically decoded messenger RNA (mRNA) and participated in protein translation. tRNA-derived fragments (tRFs), also known as tRNA-derived small RNAs (tsRNAs), are generated by the specific cleavage of pre- and mature tRNAs and are a class of newly defined functional small non-coding RNAs (sncRNAs). Following the different cleavage positions of precursor or mature tRNA, tRFs are classified into seven types, 5′-tRNA half, 3′-tRNA half, tRF-1, 5′U-tRF, 3′-tRF, 5′-tRF, and i-tRF. It has been demonstrated that tRFs have a diverse range of biological functions in cellular processes, which include inhibiting protein translation, modulating stress response, regulating gene expression, and involvement in cell cycles and epigenetic inheritance. Emerging evidences have indicated that tRFs in extracellular vesicles (EVs) seem to act as regulatory molecules in various cellular processes and play essential roles in cell-to-cell communication. Furthermore, the dysregulation of EV-associated tRFs has been associated with the occurrence and progression of a variety of cancers and they can serve as novel potential biomarkers for cancer diagnosis. In this review, the biogenesis and classification of tRFs are summarized, and the biological functions of EV-associated tRFs and their roles as potential biomarkers in human diseases are discussed.

**Supplementary Information:**

The online version contains supplementary material available at 10.1007/s00109-022-02189-0.

## Introduction

Over the past decade, advances in new technologies such as next-generation deep sequencing have led to the discovery of tens of thousands of non-coding RNAs (ncRNAs), which exist extensively in organisms and have multiple known functions. ncRNAs that are transcribed from DNA and do not translate into proteins include long ncRNAs (lncRNAs, > 200 nucleotides (nts)) and small ncRNAs (sncRNAs, < 200 nts) [1]. Small ncRNAs consist of various RNA species, including small nucleolar RNAs (snoRNAs), small nuclear RNAs (snRNAs), microRNAs (miRNAs), endogenous small interfering RNAs (endo-siRNAs), Piwi-interacting RNAs (piRNAs), snoRNA-derived small RNAs (sdRNAs), ribosomal RNA (rRNA)-derived fragments (rRFs), and tRNA-derived fragments (tRFs) [2–4]. tRNAs are one of the most abundant cellular ncRNAs discovered so far and account for 4–10% of all cellular RNA [5]. There are about 500 tRNA genes or tRNA gene-like sequences in the human genome. The recent discovery of tRNA-lookalikes has doubled this number [6]. Traditionally, tRNAs specifically recognize messenger RNA (mRNA) codons, transport amino acids to ribosomes, convert genetic information into corresponding polypeptide chains, and participate in protein translation.

Recently, fragments originating from tRNA, known as tRNA-derived fragments (tRFs), have gradually gained wider attention [4]. Similar to miRNAs, expression of some tRFs is commonly observed in cells and tissues from a diverse breadth of organisms, ranging from *E. coli* to humans [1]. tRFs are first identified in prostate carcinoma cell lines by Lee et al. [4], and later are also called tRNA-derived RNAs (tDRs) or tRNA-derived small RNA (tsRNA) [7, 8]. They are derived from tRNA precursors (pre-tRNAs) or mature tRNAs and can generally be classified into seven categories: 5′-tRNA half, 3′-tRNA half, tRF-1, 5′U-tRF, 3′-tRF, 5′-tRF, and i-tRF, following their differing lengths and cleavage positions [7, 9]. Although the cleavage of tRNA was found as early as 1958 [10], understanding the functions of these tRNA processing intermediates has been neglected until recently [11]. A growing body of studies have demonstrated that tRFs are not always by-products of random tRNA cleavage, but play crucial roles in numerous cellular biological processes, such as regulation of gene and protein expression, stress granule (SG) assembly, RNA processing, modulation of the DNA damage response, inheritance of acquired characteristics, cancer progression, and neurodegeneration [12].

Extracellular vesicles (EVs) are small, lipid membrane particles secreted by almost all kinds of normal and diseased cells into the extracellular environment in different ways. Based on the size and biogenesis, EVs are divided into three main categories: exosomes, microvesicles, and apoptotic bodies [13, 14]. The most extensively researched exosomes originate from cytoplasmic multivesicular bodies (MVBs) and are released following fusion with plasma membranes. They typically range in diameter from 30 to 150 nm and are actively secreted by a variety of living cell types, including immune, neural, muscle, epithelial, and stem cells. Microvesicles range in size from 100 nm to 1 μm and arise via outward budding from the plasma membrane. Apoptotic bodies measuring 1 to 5 μm are derived from apoptotic cells during programmed cell death [15, 16].

So far, EVs have been isolated from all human body fluids, such as blood, urine, cerebrospinal fluid, saliva, and milk [17, 18]. Since their initial definition in 1983, it is currently known that EVs are central players in intercellular communication and signaling [19, 20]. Many evidences have also demonstrated their promising functions within tumorigenic processes, immune responses, cardiovascular diseases, nervous system-related diseases, and interplay between pathogens and hosts [21]. The cargoes of EVs contain membrane proteins, cytoplasmic proteins, nuclear proteins, extracellular matrix proteins, specific lipids, and nucleic acids including DNA, mRNA, tRNAs, miRNAs, lncRNAs, circRNAs, tRFs, and ribosomal RNAs [22–25]. To promote the research on extracellular RNA (exRNA), the Extracellular RNA Communication Consortium (ERCC) has established an extracellular RNA atlas across 5 human biofluids, which integrates a diverse set of exRNA-seq and qPCR sample profiles from 19 different studies [26, 27]. Further investigation reveals 6 major exRNA cargo types in vesicle, ribonucleoprotein, and lipoprotein carriers [26].

EVs are good natural carriers of small RNAs with regulatory function between cells. MiRNAs are the most deeply studied RNA types within EVs. Work on miRNA has dominated this field; nevertheless, emerging evidence has shown that EV-associated tRFs can contribute towards signaling between cells and can also serve as potential biomarkers for diseases. However, the related mechanisms remain unclear. We suggest that the reader refers to two recent reviews by Tosar and Cayota [28] and Torres and Martí [29], which have reviewed the current descriptions of extracellular tRFs. In more recent years, tRFs have been identified in EVs from the epididymis. Some specific tRFs are conveyed from somatic cells to maturing sperm and finally to embryos [30]. This review first explores the biogenesis and classification of tRFs and then describes the biological functions of EV-associated tRFs and their potential applications as biomarkers in human diseases.

## Biogenesis of tRNAs

In the nucleus, the tRNAs are transcribed from the tRNA gene using RNA polymerase III (Pol III). The initial tRNA transcripts, also known as pre-tRNAs, have 5′-leader and 3′-tailer sequences. During tRNA maturation, the 5′-leader sequence and 3′-tailer sequence are cleaved by endonuclease P (RNase P) and endonuclease Z (RNase Z), respectively [31, 32]. Subsequently, the trinucleotide CCA is added to the 3′ end via tRNA nucleotidyltransferase to promote aminoacylation of the tRNA. Before being transported to the cytoplasm, tRNAs undergo extensive post-transcriptional modification, which further affects the structure, stability, and function of the tRNA. Mature tRNAs are70–90 nt long and fold into an L-shaped tertiary structure comprising a D-loop, an anticodon loop, a T-loop, a variable loop, an acceptor arm and a D arm, an anticodon arm, and a T arm [33].

### Types of tRFs

**Fig. 1:**
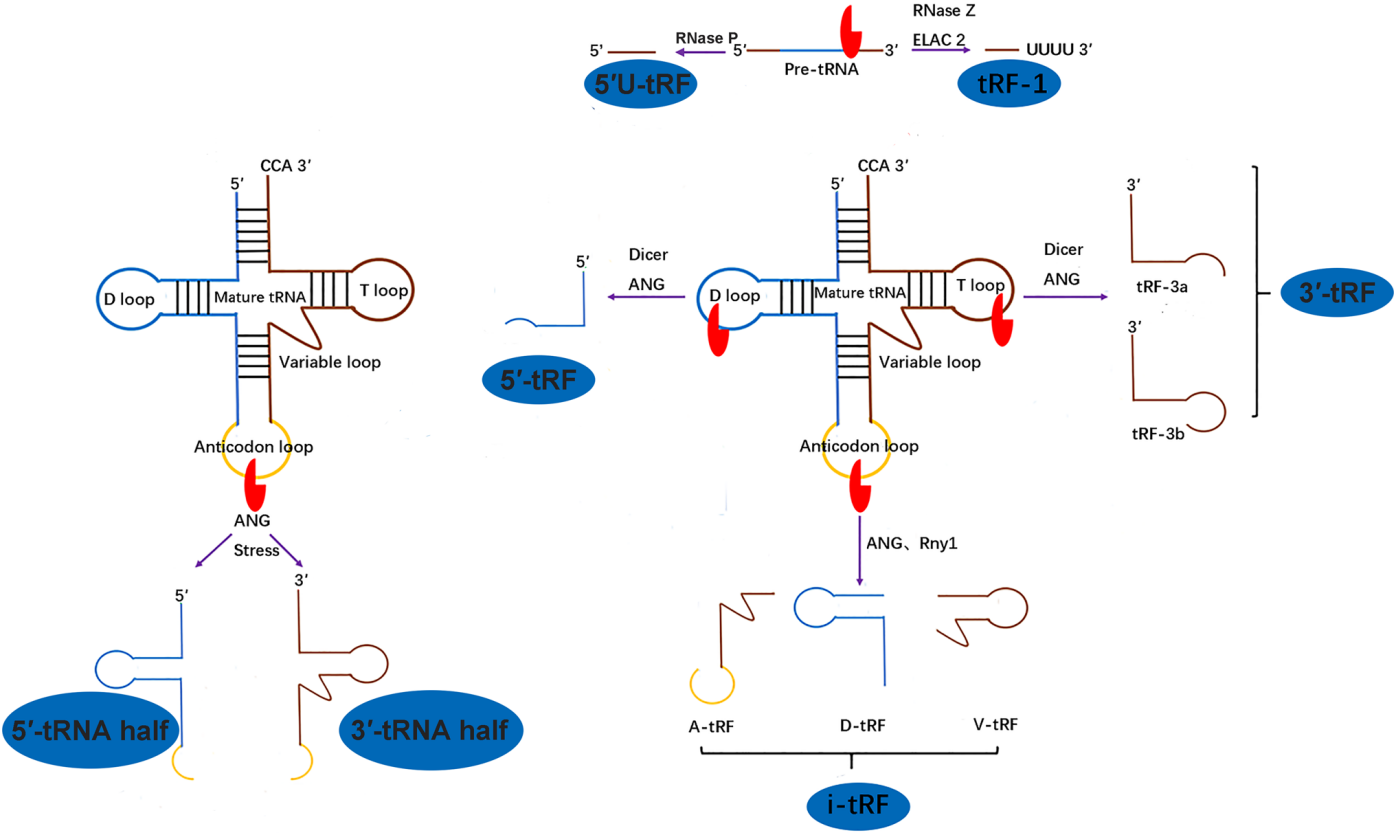
Classification of tRNA-derived fragments (tRFs). tRFs can be divided into 7 subtypes, 5′-tRNA half, 3′-tRNA half, tRF-1, 5′U-tRF, 3′-tRF, 5′-tRF, and i-tRF. tRNA half can be categorized into 2 types, 5′-tRNA half and 3′-tRNA half. They are cleaved by angiogenin (ANG) at the anticodon loop. tRF-1 is derived from precursor tRNAs digested by RNase Z or ELAC2. 5′U-tRF comes from 5′ leader of pre-tRNAs. 3′-tRF and 5′-tRF originate from mature tRNAs using ANG, Dicer, or other RNases. i‐tRF is from the internal region of mature tRNAs by ANG and Rny1

On the basis of their mapping positions on pre-tRNA or mature tRNA transcripts, tRFs can be classified into seven categories, 5′-tRNA half, 3′-tRNA half, tRF-1, 5′U-tRF, 3′-tRF, 5′-tRF, and i-tRF (Fig. 1). These tRF subclassifications can be found in organisms ranging from yeasts to humans [34].

tRNA halves are created by angiogenin (ANG, Rny1 in yeast) cleavage within anticodon loops in mature tRNAs [35–37]. Depending on whether the 5′- or 3′-sequence includes the anticodon cleavage sites, tRNA halves can be classified into two subclasses, 5′‐tRNA halves and 3′‐tRNA halves [35, 38, 39]. 5′‐tRNA halves are 30–35 nt long and initiate from the 5′ end of mature tRNAs to the anticodon loop. 3′‐tRNA halves are 40–50 nt and begin at the anti-codon loop to the 3′ end of mature tRNAs [40]. In addition, it has been reported that other RNases than ANG might also create tRNA half [41]. tRNA halves are usually produced under certain stress conditions, such as oxidative stress, hypoxia, starvation, heat shock, ultraviolet irradiation, and virus infection [35, 38].

tRF-1, 3′-tRF, and 5′-tRF are the three principal classical categories. Each unique tRF may have an identification depending on the databases developed for tRFs [42, 43]. Generally, tRF-1 series are present at a lower abundance than 3′-tRF or 5′-tRF series [34].

tRF-1s, also named 3′U tRF, is derived from the cleavage of the 3′ ends of pre-tRNAs using RNase Z in the nucleus or tRNA 3′-endonuclease ELAC2 in the cytoplasm [4, 44]. tRF-1s are generally 16–48 nt. Previous studies have indicated that tRF-1s are processed and accumulated in the nucleus and are subsequently exported to the cytoplasm [45]. This suggests that tRF-1s may play regulatory roles in some unknown steps.

5′U-tRFs originate from the 5′ leader of pre-tRNAs and most of them are 17 nt long. Certain 5′U-tRFs are obtained from the sequences just next to, or only 1 nt away from the sequence of mature tRNA, indicating that they are products of pre-tRNAs processing. These fragments have been identified in prostate cancer patient samples [10, 46].

3′-tRFs, also called tRF-3s, are generated from the 3′ ends of mature tRNAs and are produced via cleavage of Dicer, ANG, and other ribonuclease superfamily members at the TψC loop. They usually end with a universal “CCA” trinucleotide. Depending on the length of 3′-tRFs, they can be further divided into two subtypes: (1) tRF-3a, cleaved site right before the TψC loop; (2) tRF-3b, cleaved site within the TψC loop [1].

5′-tRFs, also refer to tRF-5s, originate from the 5′ ends of mature tRNAs and are cleaved at D-loop or the arm region between the D-loop and the anticodon loop in a Dicer-dependent manner. However, they can also be produced by the actions of other nucleases, such as ANG [35]. The 5′-tRFs are grossly abundant in the nucleus, while 3′-tRFs and tRF-1 s are primarily present in the cytoplasm [34, 47]. Plenty of 5′-tRFs have been identified using deep sequencing of cervical cancer HeLa cells, cleaved by Dicer with a length of 19 nt. These small tRNA fragments combine poorly with Argonaute (Ago) 1 and Ago 2 [48]. In addition, there are 5′-phosphate and 3′-hydroxyl ends in 3′-tRFs and 5′-tRFs, which are similar to miRNAs.

i‐tRFs, also referred to as internal tRFs, mainly originate from the internal regions of mature tRNAs [49]. i‐tRFs have only just been discovered and their classification is based upon their starting location at the 5′-end of tRNA. D-tRFs and A-tRFs are produced by cutting at the D stem and anticodon loop, respectively, and V-tRFs are derived from cleavage at the variable loop [50]. Moreover, i‐tRFs are highly abundant and may vary depending on gender, population, race, amino acid characteristics, anticodons, tissues, diseases, and disease subtypes [49].

In addition to above mentioned tRFs, other types of tRNA fragments have also been observed [51, 52]. Sex hormone-dependent tRNA-derived RNAs (SHOT-RNAs) are a category of tRNA half and have been discovered in sex hormone-dependent cancers. In particular, they are enriched explicitly in cell lines from estrogen receptor (ER) positive breast cancer and androgen receptor (AR) positive prostate cancers. The SHOT-RNAs are derived from ANG-mediated cleavage at the anticodon loop of aminoacylated tRNAs, promoted by sex hormone signaling pathways [53]. Thus, 5′-SHOT-RNAs have a phosphate at the 5′-terminal and a 2′,3′-cyclicphosphate at the 3′-terminal, whereas 3′-SHOT-RNAs contain a 5′-hydroxyl at the 5′-terminal and an amino acid at the 3′-terminal. tRF-2s consist of an anticodon loop and a stem structure, and the 5′ and 3′ parts of the primary or mature tRNA are excluded. They have been detected in breast cancer cells and originate from tRNA^Glu^, tRNA^Asp^, tRNA^Gly^, and tRNA^Tyr^ [54]. Schaffer et al. identified a novel 5′ leader exon generated from impaired pre-tRNA cleavage, with loss of cleavage and polyadenylation factor I subunit 1 (CLP1) kinase activity and destabilized tRNA endonuclease complex (TSEN), which correlated with a progressive loss of neurons [51]. Furthermore, Haussecker et al. identified two categories of tRFs (type I and type II tRFs) based on their cleavage enzymes. Type I tRFs are Dicer-dependent. However, type II tRFs require RNase Z to cleave pre-tRNAs in the nucleus [47].

### Nomenclature and databases of tRFs

It is vital to establish a standardized nomenclature for tRFs to facilitate academic communication and research. However, there is lack of consistency of naming system for tRFs so far. To promote research and academic exchange, scientists have created various databases of tRFs, including tRFdb (http://genome.bioch.virginia.edu/trfdb/), MINTbase (http://cm.jefferson.edu/MINTbase/), tRFexplorer (https://trfexplorer.cloud/), and others (Table 1) [7, 42, 43, 55–62]. Currently, the nomenclature of tRFs varies among different databases and researches. In tRFdb, tRFs were specified a unique ID starting with “3” (e.g., tRF-3019a, tRF-3017a), mapping at the 3′ end of mature tRNA [42, 63]. The first identified tRF-1 was named tRF-1001 according to the order of discovery [4]. A tRF is given a unique MINTbase ID (e.g., tRF-19-3L7L73JD) by the License Plate nomenclature, which are based only on tRF sequence and transcend species [43]. Additionally, differentially expressed tRFs were identified and named in small RNA libraries constructed in different studies (e.g., tRF-03357, tDR-5334) [64, 65]. In some studies, tRFs were also named by the length of the tRFs (e.g., tRF-25, tRF-18) or labeled by the authors (e.g., tRF-315, tRF-544) [46, 66].

**Table 1:** tRNA-derived fragment databases

Database name	Characteristics	URL link	Established time
tRFdb	The first database of tRFs; contains 3 types of tRFs from 8 species; provides the tRNA genome coordinates and names	http://genome.bioch.virginia.edu/trfdb/	2015
PtRFdb	A database for plant tRFs; supplies core information of 3 types of tRFs 10 plant species	http://www.nipgr.res.in/PtRFdb/	2018
tRex	The first database of tRFs in plant *Arabidopsis thaliana*; makes *Arabidopsis* tRF research very convenient	http://combio.pl/trex	2018
MINTbase 2.0	Contains 26,531 nuclear and mitochondrial tRFs from multiple human tissues; users can acquire information about maximum abundance of tRFs and their parental tRNA modifications	http://cm.jefferson.edu/MINTbase/	2018
tRF2Cancer	Facilitates users to study the expression of tRFs in multiple cancers	http://rna.sysu.edu.cn/tRFfinder/	2016
tRFexplorer	Allows users to investigate expression profile and correlation analyses of tRFs in NCI-60 cell line and TCGA tumor samples	https://trfexplorer.cloud/	2019
OncotRF	Exhibits dysregulated tRFs in cancers and their functional annotations and clinical relevance	http://bioinformatics.zju.edu.cn/OncotRF	2020
MINTmap	Very quick for users to identify tRFs and calculate the raw and normalized abundances of tRFs	https://github.com/TJU-CMC-Org/MINTmap/	2017
tDRmapper	Offers a standardized naming and quantifying scheme for tRFs; facilitates users to discover novel biology of tRFs	https://github.com/sararselitsky/tDRmapper	2015
tsRBase	Includes 121,942 tRFs by small RNA-seq data from 20 species; integrates tRF’s expression with functional characteristics	http://www.tsrbase.org	2020

Several problems lead to the difficulties in setting up a standardized naming scheme for tRFs. Challenges of obtaining the precise origin of tRFs were due to tRNA incomplete annotation, isotypes, and extensive chemical modifications. Moreover, only a few tRFs have been experimentally validated [67].

## Different abundance of EV-associated tRFs in human tissues and fluids

Some studies have revealed that the abundance of tRFs varies greatly among a variety of different tissues (Table 2) [27, 68–73]. The same parental tRNA can generate different tRFs depending on different tissues [49]. It has been found that tRNA-Gly-GCC-5–1 can produce a tRNA half in colon tissues, but generates a 5′-tRF in liver tissues and seminal fluid and a 3′-tRF in neural progenitor samples, respectively [7].

**Table 2:** Distribution of EV-associated tRFs in human tissue

Name of disease	tRF/tRNA name	Methods for EVs isolation	Sample type	EV type	Reference
Preeclampsia	tRNA-Ala-AGC	Ultracentrifugation	Human placentae	Syncytiotrophoblast-derived extracellular vesicles (STB-EV)	[68]
Normal pregnancy	5′-tRNA-half-GlyGCC	Ultracentrifugation	Human placentae	Syncytiotrophoblast-derived extracellular vesicles (STB-EV)	[69]
Healthy donors	5′-tRNA-half-Gly, 5′-tRNA-half-Val	Ultracentrifugation	Human semen	Seminal exosome (SE)	[70]
	Several tRFs	Multiple exRNA isolation methods	Human serum	EVs	[27]
	Several tRFs	Multiple exRNA isolation methods	Human plasma	EVs	[27]
Cholangiocarcinoma	Several tRFs	Multiple exRNA isolation methods	Human bile and urine	EVs	[27]
	Several tRFs	Multiple exRNA isolation methods	Cell culture conditioned medium	EVs	[27]
Cecal ligation and incision	tRNA-Gly-GCC	Ultracentrifugation	Mesenteric lymph from exemplar rat models	Mesenteric lymph extracellular vesicle (ML-EV)	[71]
Elective plastic surgery, hip replacement	tRNA-Gly-GCC	Ultracentrifugation	Human adipose tissue samples, bone marrow	Mesenchymal stem cell exosomes	[72]
Glioblastoma	Several tRFs	Ultracentrifugation	Human low-passage GBM cells	EVs	[73]

### EV-associated tRFs

To avoid degradation catalyzed by RNase in the extracellular environment, exRNAs are packaged or associated with a variety of exRNA carriers, including EVs, ribonucleoproteins (RNPs), and lipoprotein (LPP). A diverse group of small RNAs have been identified in EVs, including mRNAs, miRNAs, rRNAs, lncRNAs, tRNA fragments, circRNAs, piRNAs, and Y RNA. Recently, research into tRFs in EVs has grown, due to their regulatory functions in molecular processes and their prospects as biomarkers of disease.

#### EV-associated tRFs in blood

So far, EVs have been found in the bloodstream, including in plasma and serum. Circulating EVs can be taken up by the recipient cells and deliver signaling molecules, thereby mediating intercellular communication. In a previous study, RNA sequencing analysis of human plasma-derived exosomes indicated that tRNA accounted for only 1.24% of all mappable reads, contrasting with the most abundant of miRNAs (76.20%) [74]. Similarly, Yuan et al. detected that tRNAs occupied a small proportion (~ 2.1%) of mappable reads in RNA sequencing on plasma extracellular vesicles from a large sample [75].

Syncytiotrophoblast-derived extracellular vesicles (STB-EVs), which are released by the placenta into the maternal blood during the pregnancy, are considered to play an essential role in the adaptive changes during gestation. Wei et al. reported that the small RNAs in STB-EVs from placentae included miRNAs (60–65%), rRNAs (20.2–21.7%), and tRFs (12.8–17%) [68]. In this study, 5′-tRFs from three different tRNAs were also present at different levels between preeclamptic and normotensive trophoblast tissues from the placenta [68]. Lately, another study demonstrated that tRNA species were the most predominant (> 95%) type of short RNAs from STB-EV, in contrast to < 50% in whole placenta tissue [69]. Among these tRNA species within STB-EV, most are 5′‐tRNA halves [69]. However, Amorim et al. indicated that rRNA was reported to be the most abundant RNAs (73%) in plasma-derived EVs [76].

Generally, EV-associated tRFs seem unlikely to be the main form of tRFs in the human circulating bloodstream. Conversely, RNP, another carrier of exRNAs in the blood, has been confirmed to be highly abundant in 5′‐tRNA halves [77].

#### EV-associated tRFs in other body fluids

To date, EVs have been detected in almost all body fluids. It was identified that seminal exosomes (SE) could transmit small RNAs that serve as regulatory molecules to the recipient mucosa [70]. In a more recent study, five biofluids were compared using 10 exRNA isolation methods, including ultracentrifugation to pre-enrich the EVs. The results showed that 5′‐tRNA halves and 5′-tRFs were highly abundant in bile and urine [27]. This was consistent with the findings in the blood samples. However, 3′-tRFs and i-tRFs were more highly enriched in cells [27]. Small RNAs in mesenteric lymph (ML) may play a crucial part in critical illness. It was reported that the tRNA proportion (> 90%) in ML was more significant than those in plasma (about 45%) from sham rats and was predominantly tRNA halves 32 nt in length [71]. Among these tRNA halves, 5′‐tRNA halves from tRNA-Gly-GCC were the most abundant. Further investigation revealed that tRNA halves only occupied 1% of the ML-EV reads, whereas 76% of the total reads were miRNAs [71].

#### EV-associated tRFs in cell lines

A study on dendritic cells (DCs) demonstrated that tRFs were selectively present in EVs. Nolte-’t Hoen et al. performed small RNA analysis in both EVs and cells simultaneously [78]. Strikingly, tRFs mapped to either the 5′ or 3′ end of tRNA were both observed in the cells, whereas only 5′ fragments were highly enriched in EVs. Furthermore, most of the abundant tRFs in EVs covered regions of 40–50 nt, compared to the 30–35-nt fragments in cellular RNA. This indicated the existence of two different fragments of the same tRNA. It also confirmed that one specific tRF was observed only in EVs [78].

Another study was performed on exosomes isolated from human bone marrow- and adipose-mesenchymal stem cells (BMSCs and ASCs) [72]. It was observed that tRFs constituted the majority composition of the total tRNA in cells and exosomes. Importantly, 33-nt 5′‐tRNA halves from tRNA-Glu-CTC and tRNA-Gly-GCC were predominantly present in exosomes from ASC and BMSC. However, exosomes released by BMSC preferentially packaged the full-length tRNA-Glu-CTC and 33-nt tRNA halves of another abundant tRNA. Taken together, the different distributions of the tRFs between exosomes of bone marrow and adipose may be linked to the origins and stem of the mesenchymal stem cells (MSCs) [72]. Bioinformatics analysis revealed that several potential targets were related to the self-renewal of stem cells and MSC differentiation [72].

Research on glioblastoma (GBM) by Wei et al. exhibited that some specific 5′‐tRNA halves originated from tRNA-Gly-GCC and tRNA-Glu-CTC and showed prominence in exRNA [73]. Notably, a remarkable abundance of ANG and 5′‐tRNA half was noted in the exosomes released by GBM-derived stem-like cells that could represent therapeutic resistance, which might imply tRNA cleavage in the exosomes [73].

Another study by Sork and coworkers analyzed the diversity of RNA species within cells and EVs from five different cell types. The results revealed a remarkable richness of tRNA-Gly-GCC, tRNA-Glu-CTC, and tRNA-His-GTG, in agreement with previous research [72, 79].

Overall, many studies have indicated that tRFs are more highly enriched in EVs than those in parental cells and that they may regulate essential biological functions, particularly concerning mediating intercellular communication. In contrast, miRNAs in EVs represent a relatively small proportion, which is a different situation in cells. Interestingly, higher enrichment in non-EV or RNP fraction was observed, which is consistent with the finding in blood [73, 80].

## Biological functions of EV-associated tRFs

Much evidence has recently indicated that EV-associated tRFs play essential roles in many biological processes, particularly in regulating epigenetic inheritance, gene expression, protein synthesis, and immune activations [30, 81, 82].

### Modulating epigenetic inheritance

Some reports have shown that EV-associated tRFs are related to epigenetic inheritance in mammals. Paternal diet can influence the metabolism of subsequent generations. Research in mice exhibited that consuming a low protein diet altered the levels of multiple small RNAs in sperm [81]. 5′ fragments of tRNA-Gly-CCC, TCC, and GCC were upregulated [81]. Of note was that EVs derived from the epididymis, also known as epididymosomes, delivered 5′-tRFs to sperms and oocytes in succession. Furthermore, the authors found that tRF-Gly-GCC which was upregulated in sperm from mice fed with a low protein diet could suppress the endogenous retroelement MERVL-regulated genes in preimplantation embryos [81]. Consequently, these findings might be responsible for the metabolic alterations in offspring.

**Fig. 2:**
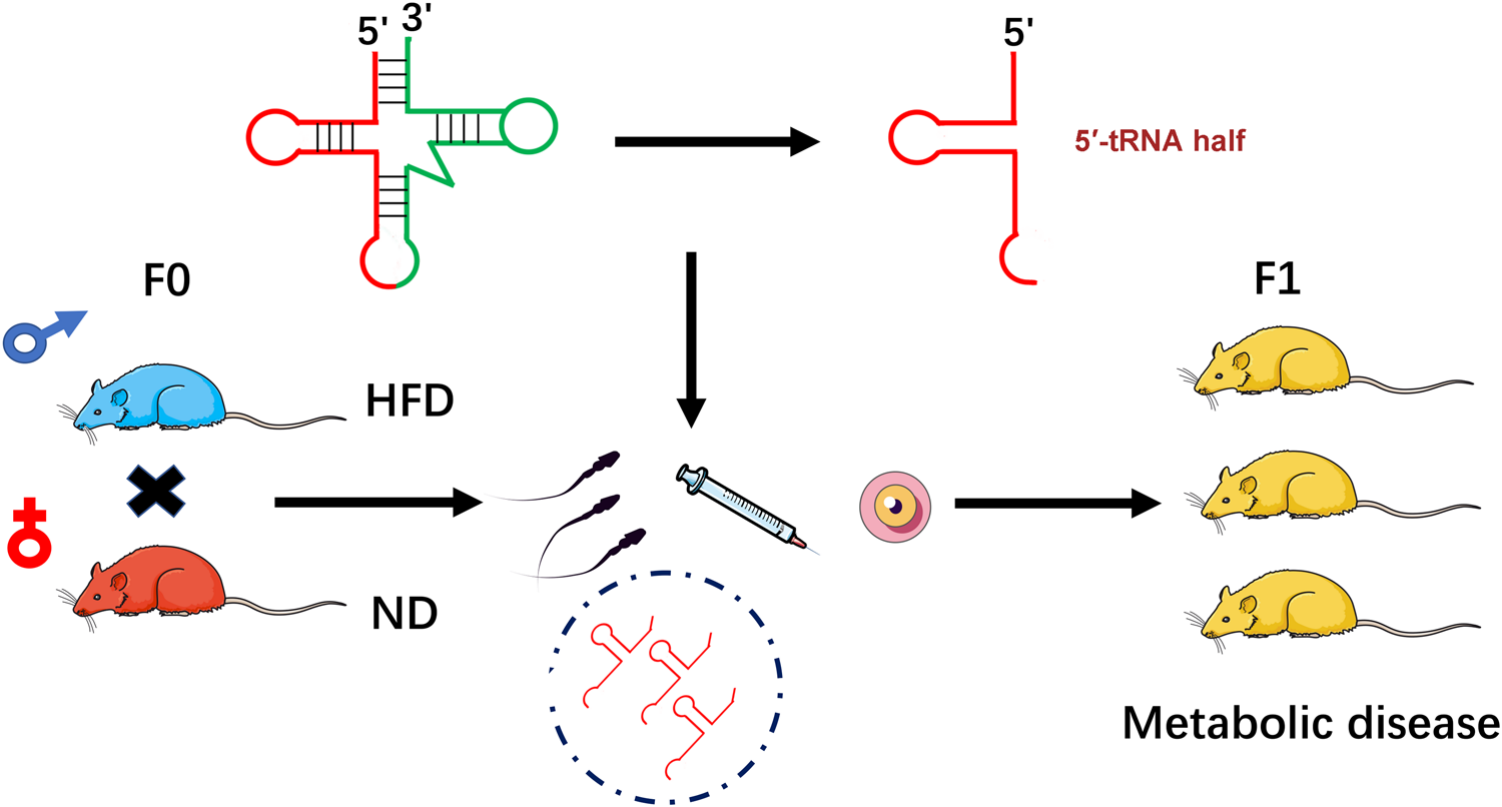
The regulation of epigenetic inheritance by 5′-tRNA halves. Epididymosomes (a type of EVs) delivered tRFs to mature sperm. 5′-tRNA halves were discovered in a paternal mouse model with high-fat diet (HFD). Injection of these tRFs generated diet-induced metabolic disorders in F1 offspring. ND, normal diet

tRFs in sperm can act as an epigenetic factor that may affect the metabolic phenotypes of future generations (Fig. 2). Chen and colleagues identified 5′‐tRNA halves in a paternal mouse model treated with a high-fat diet (HFD), which exhibited altered expression levels and RNA modifications [83]. Furthermore, they injected tRFs (30–40 nt) from the sperm of HFD mice into normal zygotes. The F1 offspring displayed changes in embryonic gene expression and RNA modifications and subsequently presented with metabolic disorders [83]. These findings demonstrated that diet-induced metabolic abnormalities could be transmitted from father to offspring, unlinked to the DNA methylation of CpG-enriched regions.

Interestingly, tRF types differ in abundance between paternal diet (low protein and high fat) in Sharma et al. [81] and in Chen et al. [83], which deserve further investigation.

### Modulating gene expression

So far, it has been reported that EV-associated tRFs might also be relevant to regulating gene expression. Sharma and coauthors noted that epididymosomes (small EVs) could deliver 5′-tRFs to caput sperm in mice [30]. tRF-Gly-GCC can repress the expression of genes related to an endogenous retroelement (MERVL) during the development of preimplantation embryos [30]. Furthermore, this specific tRF regulates the stability and activity of ncRNAs and the histone levels, consequently affecting global chromatin production [84].

EV-associated tRFs could have a significant impact on host–pathogen interactions [85]. Garcia-Silva et al. found that EVs secreted by *Trypanosoma cruzi* could induce gene expression changes in host HeLa cells [85]. Furthermore, specific transcripts were significantly changed upon transfection with two EV-associated tRFs (tRF^Thr^ and tRF^Leu^) in HeLa cells. Likewise, some of these transcripts were also affected by incubation with EVs. However, the specific mechanism of action remains to be further elucidated [85].

### Regulating protein synthesis

5′‐tRNA halves might inhibit both transcription and translation. Cooke and coauthors identified different enrichment of tRNA species between STB-EV in normal pregnancy and medium-large vesicles (MLEV). 5′-tRNA halves, not 3′-tRNA halves were the most abundant within STB-EV and confirmed by qPCR. Also, they used fibroblasts in cell cultures to investigate the effects of 5′tiRNA-GlyGCC (5′-tRNA half), which was enriched in placental STB-EV [69]. A fluorescence-labeled methionine analog quantified the global protein synthesis. Human fibroblasts showed a reduction in fluorescence by adding exogenous 5′tiRNA-GlyGCC in an in vitro model, implying that 5′-tRNA halves play an essential part in fetus-maternal signaling in normal pregnancies [69].

### Regulating immune activation

**Fig. 3:**
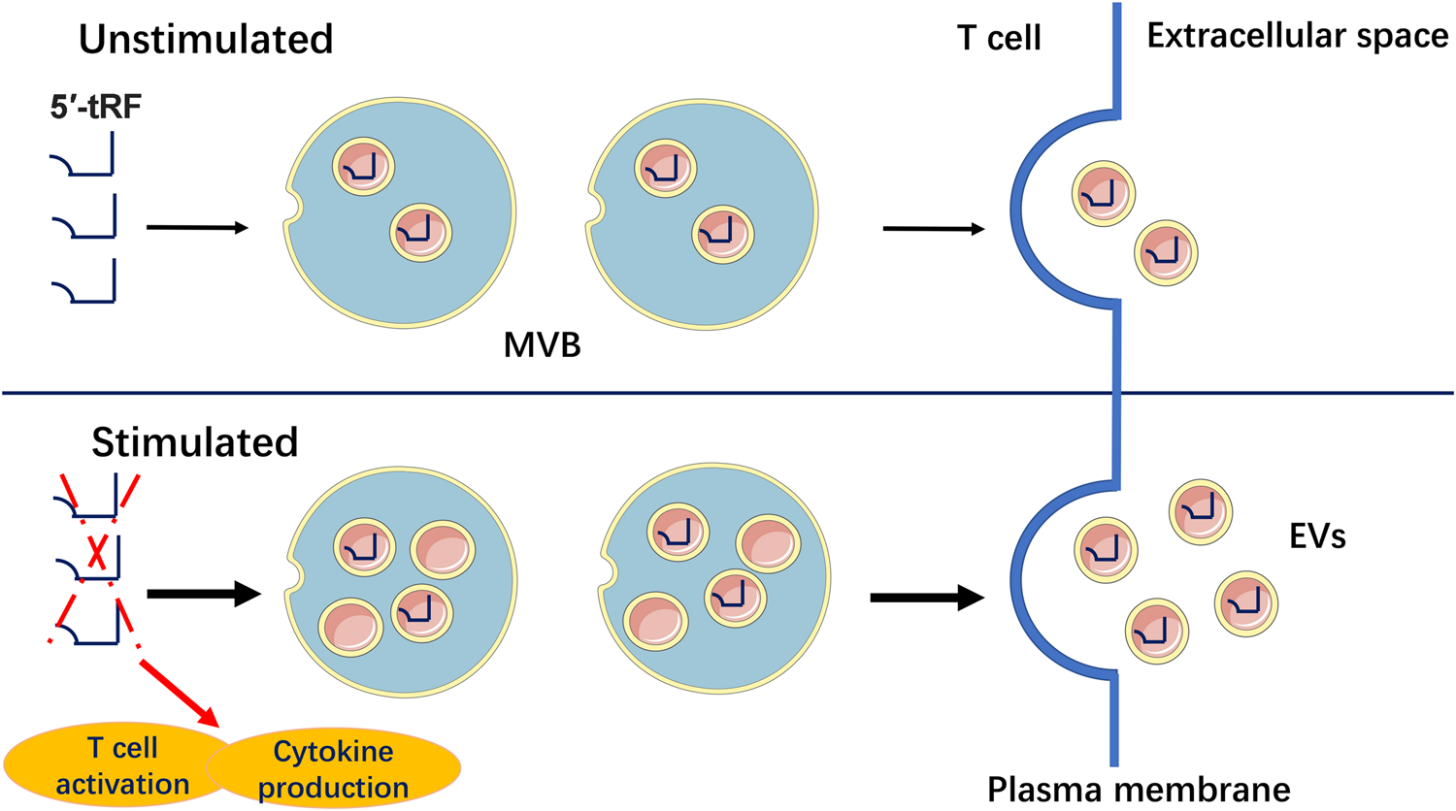
The regulation of immune activation by 5′-tRFs. T cells release 5′-tRFs into extracellular vesicles (EVs) via the multivesicular body (MVB). Immune activation signal promotion of MVB formation and the secretion of specific tRF-enriched EVs. These 5′-tRFs repress both the activation of T cells and cytokine production within T cells

Recently, a study on T cell activation was performed by Chiou’s research group [82]. The authors used a two-stage ultracentrifugation procedure to isolate EVs secreted by T cells. It was revealed that 5′-tRFs 18–21 nt in length were predominantly enriched in EVs, whereas 3′CCA-tRF measuring 17–18 and 22 nt were depleted. Further investigations found that enrichment and depletion attenuated the activation of resting T cells [82]. Compared to cellular RNA, 5′-tRFs Leu-TAA and Leu-TAG exhibited a higher abundance in EVs in the stimulated conditions, whereas 3′i-tRF Leu-TAA showed higher enrichment levels under resting conditions. These differences might contribute to the T cell response regarding activating stimulating signals. In addition, the findings demonstrated that T cell activation could downregulate the activation-induced EV-enriched tRFs in cells via MVB formation and secretion [82]. Interestingly, transfecting antisense oligonucleotides that inhibit these tRFs could promote T cell activation, suggesting that removing the activation-induced tRFs by EV-biogenesis pathways might be a key mechanism in suppressing the inhibitory effect of tRFs in T cell activation (Fig. 3).

## Clinical potential of tRFs in human diseases

Studies have revealed that tRFs were closely related and could be ideal potential biomarkers for cancer, viral infection, and metabolic and neurological diseases (Table 3) [54, 64, 65, 86–109]. Moreover, the research on EV-associated tRFs is developing rapidly, promising biomarkers for various diseases [28].

**Table 3:** Representative tRFs associated with human diseases

Disease type	Disease name	tRF/tRNA name	MINTbase Unique ID	Associated mechanisms		Reference
Cancer	Breast cancer	tRNA^Gly^, tRNA^Asp^, tRNA^Glu^, tRNA^Tyr^	Not found	Repress the stability of oncogenic transcripts by YBX1 displacement		[54]
		tDR-7816, tDR-5334, and tDR-4733	nlr-21-F5W8E7OME/tRF-18-18VBY9DV, tRF-23-NB57BK87DZ	Regulate the xenobiotic metabolic processes of oncogenesis		[65]
		tDR-0009, tDR-7336	tRF-31-P4R8YP9LON4VD/nlr-31-P4R8YP9LON4VB	Hypoxia-induced chemoresistance, modulate phosphorylation of STAT3		[86]
		tRNA-Arg, -Asn, -Cys, -Gln, -Gly, -Leu, -Ser, -Trp, and -Val, tRNA-Asp and -Lys		Biomarkers, associate with clinical characteristics		[87]
	B lymphoma	tRF-3^GlyGCC^	tRF-22-WE8SPOX52	Repress cell proliferation and regulate DNA damage response		[88]
	Cervical carcinoma	5′tDR-GlyGCC	tRF-31-P4R8YP9LON4VD	Inhibit cell apoptosis induced by cytochrome c		[89]
	Ovarian cancer	tRF5-Glu, tRF-03357, tRF-03358	Not found, tRF-29-JY7383RPD9JM, tRF-30-JY7383RPD9W1	Inhibit cell proliferation, migration and invasion		[64, 90]
	Colorectal cancer	5′-tiRNA-Val	Not found	Involve in ANG-mediated cell migration and invasion		[91]
	Gastric cancer	tiRNA-5034-GluTTC-2, tRF-19-3L7L73JD, tRF-33-P4R8YP9LON4VDP, tRF-3019a, tRF-3017a	tRF-34-86V8WPMN1E8Y2Q, tRF-19-3L7L73JD, tRF-33-P4R8YP9LON4VDP, tRF-18-8R1546D2, tRF-19-FRJ4O1E2	Regulate cell proliferation, migration and invasion		[63, 92–94, 96]
	Lung cancer	tRF‐Leu‐CAG	tRF-34-SP5830MMUKLYHE	Regulate cell cycle progression and promote proliferation in NSCLC cells		[97]
	Prostate cancer	5′-tRNA-Asp-GUC-half, 3′-tRNA-Asp-GUC-half	Not found	Disease biomarkers		[98]
		tRF-1001	nlr-20-OJR44ZIZ	Regulate cell proliferation		[4, 46]
		tRF-315, tRF-544	tRF-29-PSQP4PW3FJF4, tRF-27-87R95RM3Y82	Prognostic candidate biomarkers		
	Bladder cancer	Several tsRNAs	Not found	Intertwine with mRNAs in a sex-dependent manner		[99]
	Chronic lymphocytic leukemia	i‐tRF‐GlyGCC, i-tRF-GlyCCC, i-tRF-Phe^GAA^, tRF-Leu^AAG/TAG^	tRF-18-5J3KYU05, not found, tRF-21-ZPEK45H5D, tRF-18-HR0VX6D2	Disease biomarkers		[100–103]
	Uveal melanoma	MT tRNA-Leu-TAG, MT tRNA-Ser-GCT	tRF-22-BP4MJYSZH, tRF-21-45DBNIB9B	Associate with metastasis		[104]
Hormone-dependent cancer	Prostate cancer and breast cancer	5′‐SHOT‐RNA^AspGUC^, 5′-SHOT-RNA^HisGUG^, 5′-SHOT-RNA^LysCUU^	Not found	Enhance cell proliferation		[53]
Viral infectious diseases	Respiratory syncytial virus (RSV)	tRF5-Glu-CTC	tRF-31-87R8WP9N1EWJ0	Promote the RSV replication		[105]
	T-cell leukemia virus type 1 (HTLV-1)	tRF-3019	tRF-18-HR6HFRD2	3′-tRF	Prime HTLV-1 reverse transcription	[106]
	Chronic viral hepatitis	5′ tRH^Gly^, 5′tRH^Val^	tRF-32-PNR8YP9LON4V3, tRF-33-79MP9P9NH57SD3	5′‐tRNA halves	Abundant in chronic hepatitis B and C, altered abundance in liver cancer	[107]
Neurologic diseases	Parkinson’s disease (PD)	Several tRNAs	/	tRFs	Biomarker	[108]
	Ischemic injuries	tRNA^Val(CAC)^, tRNA^Gly(GCC)^	tRF-33-79MP9P9NH57SD3 tRF-32-PNR8YP9LON4V3	tRNA halves	Negative regulators in angiogenesis	[109]
	Amyotrophic lateral sclerosis (ALS)	TRV-AAC4-1.1 and TRA-AGC6-1.1	Not found	5′-tRF	Biomarker	[95]
	Pontocerebellar hypoplasia (PCH)	Several tRNAs	Not found	tRNA halves	CLP1 Links tRNA biogenesis to nervous system diseases	[51, 52]

### EV-associated tRFs as potential biomarkers

Most of the research on tRFs as biomarkers has mainly utilized unfractionated bloodstream with a mixed exRNA carrier. However, a growing number of studies have now focused on tRFs within EVs derived from various body fluids [22, 66, 79, 110–114] (Table 4).

**Table 4:** EV-associated tRFs as potential biomarkers

Type of disease	Name of disease	tRF/tRNA name	Methods for EVs isolation	Sample source	Reference
Cancer	Liver cancer	tRNA-Val-TAC-3, tRNA-Gly-TCC-5, tRNA-Val-AAC-5, and tRNA-Glu-CTC-5	Total exosome isolation reagent (from cell culture media), total exosome isolation kit (from plasma)	Exosomes from cell culture medium and human plasma	[22]
	Gastric carcinoma	tRF-25, tRF-38, tRF-18	/	Exosomes from human plasma	[66]
	Breast cancer	tRF-Lys-TTT	Total exosome isolation kit (Invitrogen)	EVs from cell culture medium and the human serum	[110]
	Breast cancer	tRFs (30–31 nt)	Ultracentrifugation	EVs from cell culture medium	[115]
	Breast cancer	miR-720, miR-1274b	Sequential centrifugation/ultracentrifugation	EVs from serum-free cell culture medium	[112]
Other diseases	Osteoporosis	tRF‐25‐R9ODMJ6B26, tRF‐38‐QB1MK8YUBS68BFD2, tRF‐18‐8S68BFD2	ExoQuick™ plasma prep and exosome precipitation kit	Exosomes from human plasma	[111]
	Chronic kidney disease	tRF^Val^ and tRF^Leu^	Ultracentrifugation	Exosomes from human urine	[113]
	Infection	tRNA-Leu, Thr, Glu, Gly, and Arg	Ultracentrifugation	EVs from sE48 parasite culture medium	[114]
	Male fertility	tRNA-Gln-TTG	/	Exosomes from human semen	[119, 120]

### EV-associated tRFs as biomarkers in cancer

#### Exosomal tRFs in liver cancer

Recently, Zhu et al. demonstrated the presence of tRFs in exosomes from a cultured medium of liver cancer cells [22]. Among these tRFs in exosomes, 5′-tRF was the most abundant (90%). Subsequently, 3′-tRF and i-tRF accounted for 9 and 1%, respectively [22]. In addition, the level of tRFs in plasma exosomes from patients with liver cancer was significantly higher than that from healthy controls. Following the findings in cell culture, 5′-tRF was also the predominant category of tRFs in the plasma exosomes [22]. In particular, four tRFs, tRNA-Val-TAC-3(tRF-40-EFOK8YR951K36D26, 3′-tRF), tRNA-Gly-TCC-5(tRF-34-QNR8VP94FQFY1Q, 5′-tRNA half), tRNA-Val-AAC-5(tRF-32-79MP9P9NH57SJ, 5′-tRNA half), and tRNA-Glu-CTC-5(tRF-31-87R8WP9N1EWJ0, 5′-tRF), from plasma exosomes were remarkably expressed in liver cancer patients, suggesting their potential value in cancer diagnosis [22].

#### Exosomal tRFs in gastric carcinoma

Gastric carcinoma (GC) is one of the most prevalent cancers caused by gene-environment interaction. Lin et al. identified higher plasma exosomal expression levels of tRF-25(tRF-25-DWY2MJ8F81, i-tRF), tRF-38(tRF-38-QB1MK8YUBS68BFD2, 3′-tRF), and tRF-18(tRF-18-8S68BFD2, 3′-tRF) in GC patients than healthy controls [66]. Besides this, the plasma exosomal of these three tRFs exhibited better diagnostic accuracy for GC detection by receiver operating characteristic (ROC) analyses [66].

#### EV-associated tRFs in breast cancer

Several studies have shown that tRFs may function as potential biomarkers in breast cancer (BC). Koi and associates confirmed that the expression levels of miR-23a-3p, isomiR of miR-21-5p, and tRF-Lys-TTT (tRF-32-PS5P4PW3FJHP1, 5′-tRNA half) were significantly elevated in BC compared to controls [110]. The model based on these three small RNAs demonstrated high diagnostic accuracy, the area under ROC (AUC) value reached 0.92, and could successfully distinguished stage 0 BC from cancer-free individuals [110]. Furthermore, small RNAs in EVs (mainly exosomes) were isolated from serum and cell culture media and were evaluated. Two miRNAs of the above three small RNAs were present in EVs from serum, with a significantly high expression level in BC. However, there was no significant difference in expression of tRF-Lys-TTT [110].

Similarly, several studies indicated that expression difference of EV-associated tRFs could be detected in BC cell lines. IsomiR of miR-21-5p and miR-23a-3p were more abundant in the EVs from BC cell media, while the expression levels of tRF-Lys-TTT were lower in the EVs than those of normal human breast epithelial telomerase immortalized cells [110]. Tosar and coauthors found that 5′-tRNA halves were significantly enriched in the extracellular spaces derived from the BC cell line MCF-7 compared with intracellular fractions [79]. In contrast, miRNAs presented at very low abundances in extracellular fractions [79]. Similarly, “miRNA-like” tRNA fragments (miR-720 and miR-1274b) were greatly expressed in the MCF-7 EVs uniquely and could not been found in cellular profiles [112]. These showed overexpression of tRNAs in MCF7 cells and an effective export process of tRFs. Consequently, these selected tRFs were amplified in EVs. Moreover, the study suggested a potential way to use high levels of tRFs combined with known tumor miRNAs to identify circulating tumor-derived EVs from EVs derived from other cellular fractions [112]. Furthermore, another study demonstrated that 5ʹ tiRNA-Gly (5′-tRNA half) could be secreted into MCF-7 EVs in a concentration-dependent fashion and closely replicated levels of EVs and the recipient cells [115].

### EV-associated tRFs as biomarkers in other diseases

#### Exosomal tRFs in osteoporosis

Osteoporosis is a disorder characterized by decreased bone mineral density and microarchitectural deterioration, which results in an increased risk of fracture. In a study on plasma exosomes, Zhang et al. identified 11 upregulated tRFs and 18 downregulated tRFs in osteoporosis compared with healthy controls, using small RNA sequence of plasma exosomes [111]. In addition, six categories of tRFs were included in the osteoporosis and healthy control groups, namely, 5′-tRNA half, 3′-tRNA half, tRF-1, 3′-tRF, 5′-tRF, and i-tRF [111]. Furthermore, the expression levels of tRF‐25‐R9ODMJ6B26 (3′-tRF), tRF‐38‐QB1MK8YUBS68BFD2 (3′-tRF), and tRF‐18‐BS68BFD2 (3′-tRF) were significantly higher in osteoporosis samples compared to controls [111]. In this study, a tRF panel including the above three tRFs was developed, with higher sensitivity and specificity for diagnosing osteoporosis than a single tRF [111].

#### Exosomal tRFs in chronic kidney disease

Urine may be used as a promising biomarker due to its noninvasive collection. Khurana et al. found 30 differentially expressed urinary exosomal ncRNAs in chronic kidney disease (CKD) patients using a novel computational algorithm of RNA-seq [113]. Among these exRNAs, tRF^Val^ and tRF^Leu^, originating from 5′-ends of tRNAs, were significantly less abundant in CKD patients than controls [113]. The increased expression levels of tRFs in exosomes from healthy controls may reflect an effective process to remove cellular waste from cells, which might impair kidney cells in during CKD [113].

#### EV-associated tRFs in infection

Several studies have indicated that some EV-associated tRFs were also related to infections [85, 116]. Garcia-Silva and coauthors demonstrated that *Trypanosoma cruzi* epimastigotes excreted vesicles which carry tRFs and Ago, distinctive to trypanosomatids (TcPIWI-tryp), to extracellular medium under nutrient starvation [114]. A portion of these molecules in EVs were transferred between parasites and to infection susceptible mammalian cells [114]. Ghosal et al. analyzed complements of exRNAs from *E. coli* and found that the major constituents were tRFs, not full-length tRNAs [117]. Likewise, EV-associated tRFs were identified in mouse serum infected with *Schistosoma mansoni* [118].

#### EV-associated tRFs in male fertility

Recently, it was demonstrated that EV-associated tRFs in sperm played notable roles in preimplantation embryo development, by modulating cell cycle–associated genes and retrotransposons [119]. Additionally, Chen et al. indicated that human sperm tRF derived from tRNA-Gln-TTG could affect activation of the embryonic genome via regulation of ncRNAs. Their findings showed that this specific tRF group with 30, 31, 32, 33, and 36 nt could act as a promising diagnostic biomarker and therapeutic target for male infertility [120].

## Conclusions

It has been demonstrated that tRFs play vital roles in the development and progress of multiple diseases, particularly cancers. Considerable evidence suggests that tRFs can regulate gene expression, gene translation, epigenetic inheritance, and the cell cycle. Meanwhile, an accumulating body of evidence has confirmed the abundant existence of tRFs in EVs. EV-associated tRFs might lead a key part in intercellular communication and serve as novel biomarkers for diagnosing cancer and other diseases. However, several limitations need to be considered. Firstly, the generation mechanism of tRFs and encapsulation in EVs remains to be fully elucidated. Secondly, more studies should be undertaken to confirm the diagnostic roles of EV-associated tRFs in different types of tumors and other diseases. Thirdly, the correlation of EV-associated tRFs with cancer therapy and cancer prognosis requires further investigation. Fourthly, methodological details for EV isolation vary between different studies, explaining the discrepancy in results and poor reproducibility. However, with the progression of technologies, we believe that EV-associated tRFs will play increasingly important roles in the diagnosis and treatment of human diseases.

## Supplementary Information

Below is the link to the electronic supplementary material.

Supplementary file1 (PDF 239 kb)

## Abbreviations

tRNAs: Transfer RNAs
mRNA: Messenger RNA
sncRNAs: Small non-coding RNAs
tRFs: TRNA-derived fragments
EVs: Extracellular vesicles
ncRNAs: Non-coding RNAs
lncRNAs: Long ncRNAs
sncRNAs: Small ncRNAs
snoRNAs: Small nucleolar RNAs
snRNAs: Small nuclear RNAs
exRNAs: Extracellular RNAs
miRNAs: MicroRNAs
endo-siRNAs: Endogenous small interfering RNAs
piRNAs: Piwi-interacting RNAs
sdRNAs: SnoRNA-derived small RNAs
rRFs: Ribosomal RNA (rRNA)-derived fragments
tsRNA: Transfer RNA (tRNA)-derived small RNA
tDRs: TRNA-derived RNAs
MVBs: Multivesicular bodies
ERCC: The Extracellular RNA Communication Consortium
Pol III: Polymerase III
pre-tRNAs: Precursor tRNAs
RNase P: Endonuclease P
RNase Z: Endonuclease Z
ANG: Angiogenin
Ago: Argonaute
CLP1: Cleavage and polyadenylation factor I subunit 1
TSEN: TRNA endonuclease complex
SHOT-RNAs: Sex hormone-dependent tRNA-derived RNAs
RNPs: Ribonucleoproteins
LPP: Lipoprotein
STB-EV: Syncytiotrophoblast-derived extracellular vesicle
SE: Seminal exosome
ML: Mesenteric lymph
DCs: Dendritic cells
BMSC: Bone marrow stem cells
ASC: Adipose mesenchymal stem cells
MSCs: Mesenchymal stem cells
GBM: Glioblastoma
HFD: High-fat diet
TNBC: Triple-negative breast cancer
STAT3: Signal transducer and activator of transcription 3
NSCLC: Non‐small cell lung cancer
HGSOC: High-grade serous ovarian cancer
CRC: Colorectal cancer
PD: Parkinson’s disease
CSF: Cerebrospinal fluid
PLS-DA: Partial least squares discriminant analysis
OGD: Oxygen-glucose deprivation
RSV: Respiratory syncytial virus
GC: Gastric carcinoma
ROC: Receiver operating characteristic
BC: Breast cancer
AUC: The area under ROC
CKD: Chronic kidney disease
