# Supplementary material for: Extracellular vesicles-associated tRNA-derived fragments (tRFs): biogenesis, biological functions, and their role as potential biomarkers in human diseases
Source: J Mol Med (Berl). 2022 Mar 24;100(5):679–95. doi: 10.1007/s00109-022-02189-0 (PMC9110440; doi:10.1007/s00109-022-02189-0)
Supplement: Supplementary file 1 — Supplementary file1 (PDF 239 kb) [file 109_2022_2189_MOESM1_ESM.pdf]

Supplementary Table 1 The sequence of some tRFs.

tRF ID	mintbase_ID	tRNA	Alignment Sequence	type
tDR-7816		tRNA-Gln-CTG-3-1	AGCACTCTGGACTCTGAATCT	i-tRF
tDR-5334	tRF-18-18VBY9DV	tRNA-Gly-CCC-5-1	AGTGGTAGAATTCTCGCC	i-tRF
tDR-4733	tRF-23-NB57BK87DZ	tRNA-Phe-GAA-2-1	CTGAAGATCTAAAGGTCCCTGGT	i-tRF
tDR-0009	tRF-31-P4R8YP9LON4VD	tRNA-Gly-GCC-1-1	GCATGGGTGGTTCAGTGGTAGAATTCTCGCC	5'-tRNA half
tDR-7336		tRNA-Gly-GCC-1-2	GCATGGGTGGTTCAGTGGTAGAATTCTCGCA	5'-tRNA half
tRF-03357	tRF-29-JY7383RPD9JM	tRNA-Gly-GCC	CATTGGTGGTTCAGTGGTAGAATTCTCGC	i-tRF
tRF-03358	tRF-30-JY7383RPD9W1	tRNA-Gly-GCC	CATTGGTGGTTCAGTGGTAGAATTCTCGCC	i-tRF
tRNA-ValTAC-3	tRF-40-EFOK8YR951K36D26	tRNA-Val-TAC-3	ACGCAGAAGGTCCTGGGTTCGAGCCCCAGTGGAACCACC	3'-tRF
tRNA-GlyTCC-5	tRF-34-QNR8VP94FQFY1Q	tRNA-Gly-TCC-5	GCGTTGGTGGTATAGTGGTGAGCATAGCTGCCTT	5'-tRNA half
tRNA-ValAAC-5	tRF-32-79MP9P9NH57SJ	tRNA-Val-AAC-5	GTTTCCGTAGTGTAGTGGTTATCACGTTCGCC	5'-tRNA half
tRNA-GluCTC-5	tRF-31-87R8WP9N1EWJ0	tRNA-Glu-CTC-5	TCCCTGGTGGTCTAGTGGTTAGGATTCGGCG	5'-tRF
tRF-Lys	tRF-32-PS5P4PW3FJHP1	tRNA-Lys-TTT	GCCCGGATAGCTCAGTCGGTAGAGCATCAGAC	5'-tRNA half
	tRF-25-R9ODMJ6B26	tRNA-Val-AAC, tRNA-Val-CAC	GGTTCGAAACCGGGCGGAAACACCA	3'-tRF
	tRF-38-QB1MK8YUBS68BFD	tRNA-Val-AAC, tRNA-Val-CAC	GCGAAAGGTCCCCGGTTCGAAACCGGGCGGAAACACCA	3'-tRF
	tRF-18-BS68BFD2	tRNA-Val-AAC, tRNA-Val-CAC	AACCGGGCGGAAACACCA	3'-tRF
tRF-315	tRF-29-PSQP4PW3FJF4	tRNA-Lys-CTT	GCCCGGCTAGCTCAGTCGGTAGAGCATGG	5'-tRF
tRF-544	tRF-27-87R95RM3Y82	tRNA-Phe-GAA	TCCCTGGTTCGATCCCGGGTTTCGGCA	i-tRF
tRF-25	tRF-25-DWY2MJ8F81	tRNA-Phe-GAA	AATGTTTAGACGGGCTCACATCACC	i-tRF
tRF-38	tRF-38-QB1MK8YUBS68BFD	tRNA-Val-CAC,tRNA-Val-AAC	GCGAAAGGTCCCCGGTTCGAAACCGGGCGGAAACACCA	3'-tRF
tRF-18	tRF-18-8S68BFD2	tRNA-Val-CAC	TCCCGGGCGGAAACACCA	3'-tRF
tRF-3^{GlyGCC}	tRF-22-WE8SPOX52	tRNA-Gly-GCC	TCGATTCCCGGCCAATGCACCA	3'-tRF
5'tDR-GlyGCC	tRF-31-P4R8YP9LON4VD	tRNA-Gly-GCC	GCATGGGTGGTTCAGTGGTAGAATTCTCGCC	5'-tRNA half
tiRNA-5034-GluTTC-2	tRF-34-86V8WPMN1E8Y2Q	tRNA-Glu-TTC	TCCCATATGGTCTAGCGGTTAGGATTCCTGGTTT	5'-tRNA half
	tRF-19-3L7L73JD	tRNA-Val-AAC	CCGTAGTGTAGTGGTTATC	i-tRF
	tRF-33-P4R8YP9LON4VDP	tRNA-Gly-GCC	GCATGGGTGGTTCAGTGGTAGAATTCTCGCCTG	5'-tRNA half
tRF-3019a	tRF-18-8R1546D2	tRNA-Ala-AGC-1-1	TCCCCAGTACCTCCACCA	3'-tRF
tRF-3017a	tRF-19-FRJ4O1E2	tRNA-Val-TAC	AGCCCCAGTGGAACCACCA	3'-tRF
tRF-1001		tRNA-Ser-TGA	GAAGCGGGTGCTCTTATTTT	
tRF-Leu-CAG	tRF-34-SP5830MMUKLYHE	tRNA-Leu-CAG1, tRNA-Leu-CAG	GTCAGGATGGCCGAGCGGTCTAAGGCGCTGCGTT	5'-tRNA half
i-tRF-GlyGCC	tRF-18-5J3KYU05	tRNA-Gly-GCC	GAGGCCCGGGTTCGATTC	i-tRF
i-tRF-Phe^{GAA}	tRF-21-ZPEK45H5D	trnaMT_PheGAA_MT_+_577_647	TTTAGACGGGCTCACATCACC	i-tRF
tRF-Leu$^{AAG/TAG}$	tRF-18-HR0VX6D2	tRNA-Leu-AAG	ATCCCACCGCTGCCACCA	3'-tRF
tRF5-GluCTC	tRF-31-87R8WP9N1EWJ0	tRNA-Glu-CTC	TCCCTGGTGGTCTAGTGGTTAGGATTCGGCG	5'-tRF
tRF-3019	tRF-18-HR6HFRD2	tRNA-Pro-AGG	ATCCCGGACGAGCCCCCA	3'-tRF
tRNA$^{Val(CAC)}$,5'tRH^{Val}	tRF-33-79MP9P9NH57SD3	tRNA-Val-CAC,tRNA-Val-AAC	GTTTCCGTAGTGTAGTGGTTATCACGTTCGCCT	5'-tRNA half
tRNA$^{Gly(GCC)}$,5' tRH^{Gly}	tRF-32-PNR8YP9LON4V3	tRNA-Gly-GCC	GCATTGGTGGTTCAGTGGTAGAATTCTCGCCT	5'-tRNA half

5'-tRNA-half-GlyGCC	tRF-31-PNR8YP9LON4VD	tRNA-Gly-GCC	GCATTGGTGGTTCAGTGGTAGAATTCTCGCC	5'-tRNA half
tRNA-Gly-GCC	tRF-33-P4R8YP9LON4VDP	tRNA-Gly-GCC	GCATGGGTGGTTCAGTGGTAGAATTCTCGCCTG	5'-tRNA half
